# A Changing World in Gene Therapy Research: Exciting Opportunities for Medical Advancement and Biosafety Challenges

**DOI:** 10.1089/apb.2021.0020

**Published:** 2021-11-24

**Authors:** Daniel Eisenman, Shaun Debold, James Riddle

**Affiliations:** Advarra, Columbia, Maryland, USA.

**Keywords:** gene therapy, Institutional Biosafety Committee (IBC), NIH guidelines, Food and Drug Administration (FDA), investigational new drug (IND)

## Abstract

**Introduction:** We previously reported on the United States' regulatory environment evolving to accommodate an emerging boom in gene therapy research. Several important developments have transpired in the 2 years since that article was published, including the coronavirus disease 2019 (COVID-19) pandemic and the drive for large-scale testing of vaccines containing recombinant or synthetic nucleic acid molecules. This report highlights key developments in the field with a focus on biosafety and issues of note to biosafety professionals with responsibilities over clinical research.

**Discussion:** We provide guidance for performing risk assessments on the currently approved gene therapy products as well as the most utilized types of investigational products in clinical trials. Areas of focus include the prominent approaches utilized in the three major areas of research: oncology, infectious diseases, and rare diseases.

**Conclusion:** The COVID-19 pandemic has created several opportunities for continued growth in gene therapy. National vaccination campaigns will result in greater public acceptance of gene therapy research. Technological advancements that made the vaccine race possible will spur the next generation of research. Advancements born in the developed world set the stage for the creation of therapeutics to treat greater numbers in the developing world and have the potential for massive benefits to global public health. Biosafety professionals and Institutional Biosafety Committees play key roles in contributing to the safe evidence-based advancement of gene therapy research. Biosafety professionals responsible for clinical research oversight must be aware of emerging technologies and their associated risks to support the safe and ethical conduct of research.

## Introduction

We previously reported on the United States' regulatory environment evolving to accommodate an emerging boom in gene therapy research.^1^ In that study, we reported on factors contributing to the surge in gene therapy research despite earlier setbacks at the turn of the century. Researchers focused on improving the wealth of knowledge on gene transfer technology and redesigning vectors for increased safety along with incorporating additional safeguards as recommended by Food and Drug Administration (FDA) guidance. The National Institutes of Health (NIH) Office of Science Policy (OSP) sequentially loosened the regulatory requirements for gene therapy research over subsequent versions of NIH guidelines as the regulatory oversight focus shifted to the FDA.^2,3^

As the FDA is tasked with assessing the safety and efficacy of therapeutic products and regulating their approval, it is a logical progression for that agency to have an increased role in overseeing clinical trials involving recombinant or synthetic nucleic acid molecules as the field matures and the technology becomes mainstream. The FDA took several steps to aid this transition, including the creation of various guidance documents regarding the manufacture of gene therapy products as well as the design of clinical trials for such products. Although several tracks already exist for prioritized review of investigational new drugs (INDs), the FDA created the Regenerative Medicine and Advanced Therapies designation to allow for expedited review of biologics, such as “gene therapies, including genetically modified cells, which lead to a durable modification of cells or tissues,” specifically used to treat serious or life-threatening diseases where preliminary clinical evidence indicates that the drug has the potential to address unmet medical needs.^4^

## The Coronavirus Disease 2019 Pandemic Aided in the Transition Toward Clinical Use of Recombinant DNA Becoming Mainstream

Within the past decade, use of recombinant or synthetic nucleic acid molecules in clinical trials has gone from limited to early phase trials utilizing small numbers of major academic medical centers to large late phase trials with each utilizing tens or hundreds of sites across the United States and globally. As of this writing, all coronavirus disease 2019 (COVID-19) vaccines with FDA emergency use authorization were produced with recombinant DNA technology containing either messenger RNA (mRNA) derived from recombinant plasmids or a recombinant adenovirus as the active ingredient. To date, >300 million doses have been administered in the United States as the population strives toward herd immunity.^5^ COVID-19 vaccine are currently administered at community vaccination sites, chain pharmacies, and even athletic stadiums.^6–8^

Since our earlier report, several major developments have transpired within the field of gene therapy research with significance to biosafety and biosafety professionals with responsibilities over clinical trials. The highlights are outlined here with a focus on the changing regulatory environment, emerging science, and the biosafety challenges they represent.

## The Number of IND Applications for Gene Therapy Products Has Remained Strong Despite COVID-19

**Figure 1.**
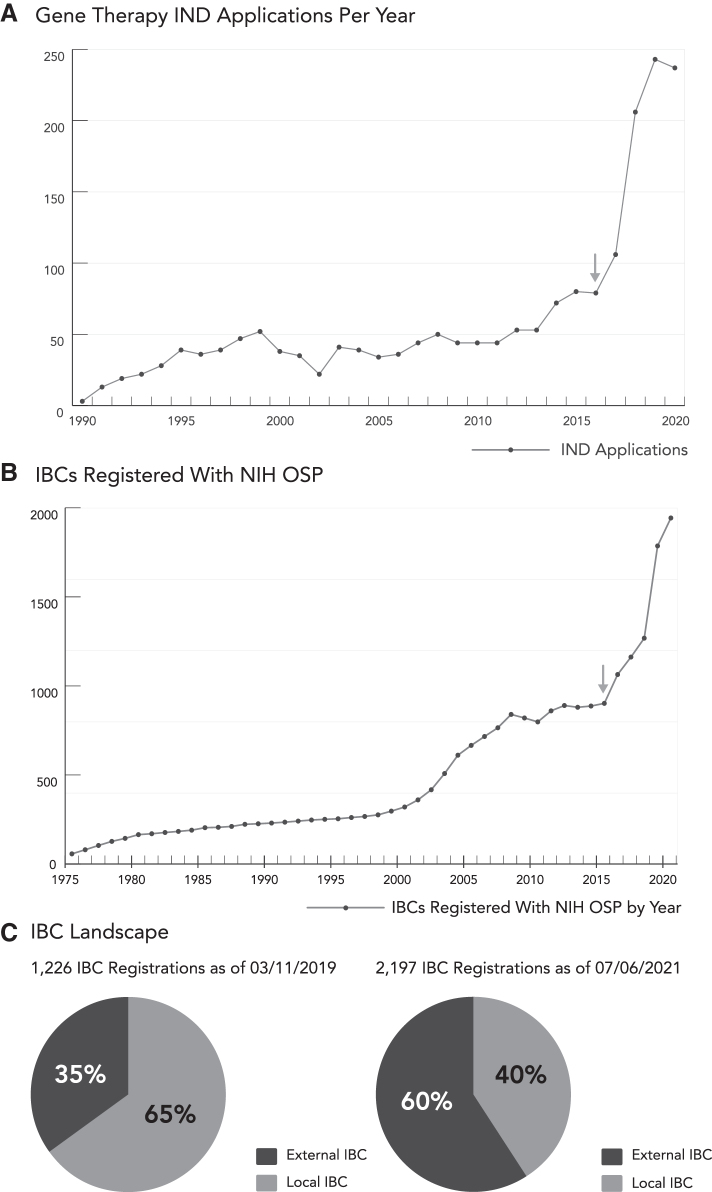
Gene therapy research caused a growth in IBCs registered with the NIH. **(A)** Gene therapy IND applications submitted per year to the FDA. Data adapted with permission from Peter Marks, Director, FDA CBER. **(B)** IBC registrations approved by the NIH OSP per year. Data adapted with permission from Kathryn Harris, NIH OSP. **(C)** Change in local versus externally administered IBCs registered with NIH OSP. CBER, Center for Biologics Evaluation and Research; FDA, Food and Drug Administration; IBCs, Institutional Biosafety Committees; IND, investigational new drug; NIH, National Institutes of Health; OSP, Office of Science Policy.

The number of IND applications submitted to the FDA for gene therapy products set records each year from 2017 to 2019 (Figure 1A).^9^ The data plateaued in 2020 as the COVID-19 pandemic caused unprecedented widescale disruptions in the clinical research sector.^10–12^ The disruption was most severe in April 2020 when the number of non-COVID-19-related new trials was 50% of the number reported in January 2020 (pre-COVID-19). April 2020 also set the high-water mark for reported trial suspensions on clinicaltrials.gov with >1000 clinical trial suspensions citing COVID-19 as the principle cause.^3^ When discussing the state of gene therapy research during COVID-19, Peter Marks, Director of the FDA Center for Biologics Evaluation and Research (CBER) stated, “Although the 2020 [gene therapy IND] number is essentially flat, that flatness needs to be interpreted in the setting of COVID-19. I actually take the number of submissions during this COVID-19 time to be somewhat remarkable.”^13^

## Gene Therapy Research Drove a Surge in Institutional Biosafety Committee Registrations with the NIH

The inflection point in IND submissions to the FDA took place in 2016 (Figure 1A, arrow), which also coincided with a similar inflection point in the number of Institutional Biosafety Committee (IBC) registrations submitted to the NIH OSP (Figure 1B, arrow).^14^ As gene therapy studies progress to later phase trials, the studies require larger numbers of research subjects meeting the inclusion and exclusion criteria of the various trials, which inevitably requires larger numbers of research sites.

As later phase studies searched beyond major academic medical centers for sites with pools of research subjects with the diseases being studied, smaller research sites without existing IBCs were inevitably required. This phenomenon drove a surge of IBC registrations for clinical research sites utilizing commercial services to externally administer IBC reviews (Figure 1C). Between 2019 and 2021, externally administered IBCs went from a minority (35%) to the majority of IBCs (60%) registered with NIH OSP.^15^

Independently administered IBCs are following in the footsteps of the rapid growth of independent central Institutional Review Boards (IRBs) in the 1980s and 1990s. As more clinical research moved from the halls of academia into small private research sites without their own research oversight structures there was a growing need for an independent option that sites could use. Various independent IRBs grew rapidly in the 1980s and 1990s during a time where late phase clinical trials were moving into privately owned small research sites. Independently administered IRBs, where a commercial entity administers the board reviewing research, demonstrated several benefits in the form of faster study initiation, greater consistency, and more effective review of multisite research.

By 2006 the transition to commercially administered IRBs was proving so successful that the FDA issued specific guidance to industry on how to work with central IRBs and academic centers who used to be the nucleus for IRB review determined use of an independent commercially administered IRB improved study start-up time even within the academic setting.^16–18^ The central IRB review concept was considered so beneficial that the NIH instituted a single IRB policy for NIH funded studies (effective January 25, 2018). The Federal government followed suit with revisions to the Common Rule (45 CFR 46), the rule of ethics in the United States regarding biomedical and behavioral research involving human subjects, to require a single IRB of record for all federally funded research (effective January 20, 2020).

The NIH allows institutions to utilize multiple IBCs, creating a situation where an institution can rely on an externally administered IBC with reviewer expertise in the clinical environment and human gene transfer research, whereas retaining their locally administered IBC for basic science research involving laboratories and animal models.^19^

If we want a glimpse at the future of IBC review for multisite clinical trials we only need to look at the transition from local to independent IRB administration for the past 20+ years. Having a single entity perform the reviews poses several potential benefits in the form of faster study initiation, greater consistency in reviews across research sites, and easier management and regulatory oversight of multisite research. Likely as more gene therapy trials enter later phase, the need for and utilization of independent commercially administered IBCs will increase in kind.

## The FDA Is Approving Diverse Types of Products Containing Recombinant or Synthetic Nucleic Acid Molecules

The FDA approved the first product containing recombinant DNA in 2015, an oncolytic herpesvirus approved for use in melanoma. Since then the FDA has issued 15 approvals for therapeutics containing recombinant or synthetic nucleic acid molecules. Table 1 lists the FDA-approved products, describes the recombinant or synthetic nucleic acid molecules and provides risk information from the FDA-approved package inserts.^20–33^

**Table 1.** Food and Drug Administration-approved products containing recombinant or synthetic nucleic acid molecules

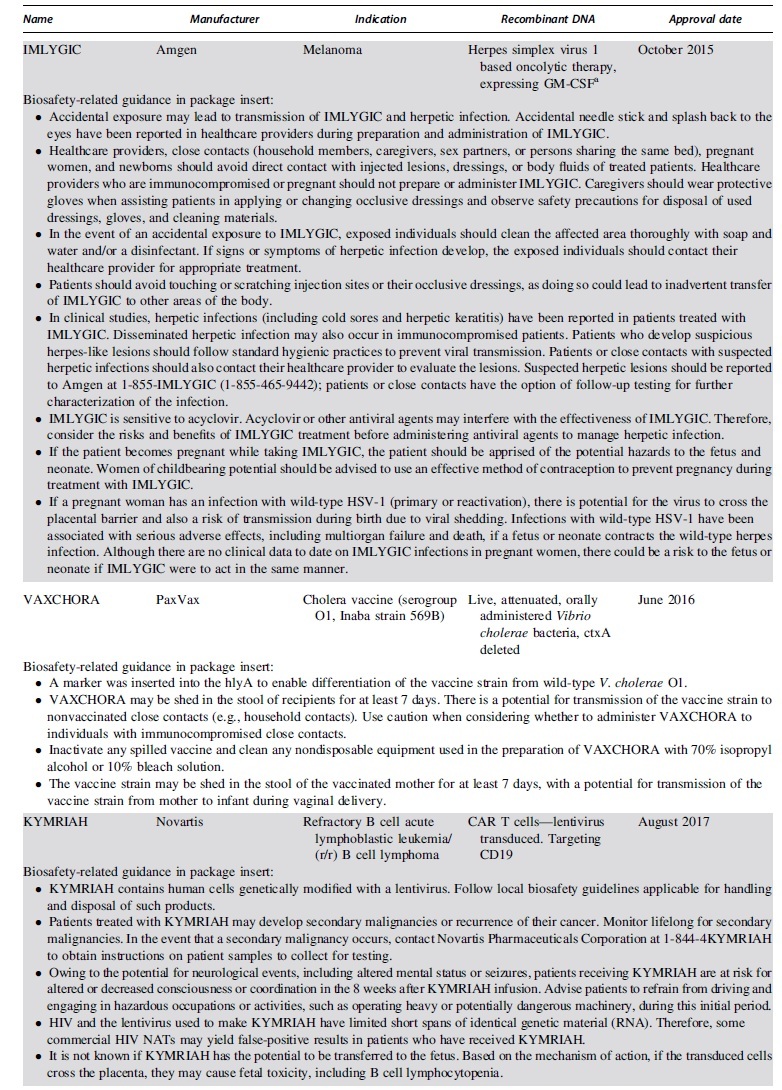 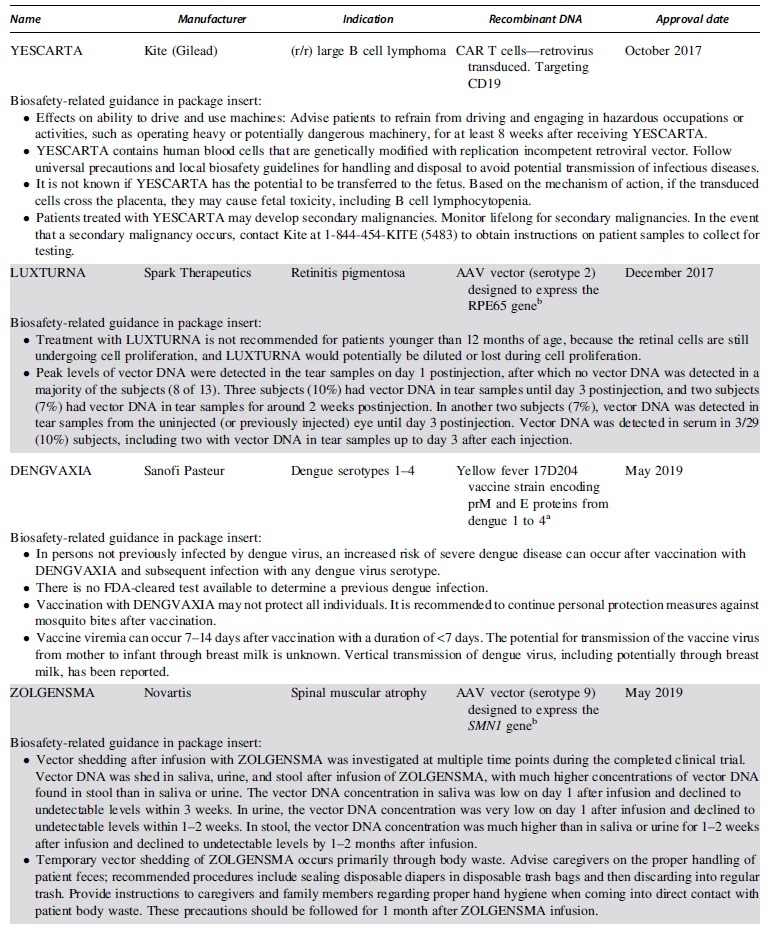 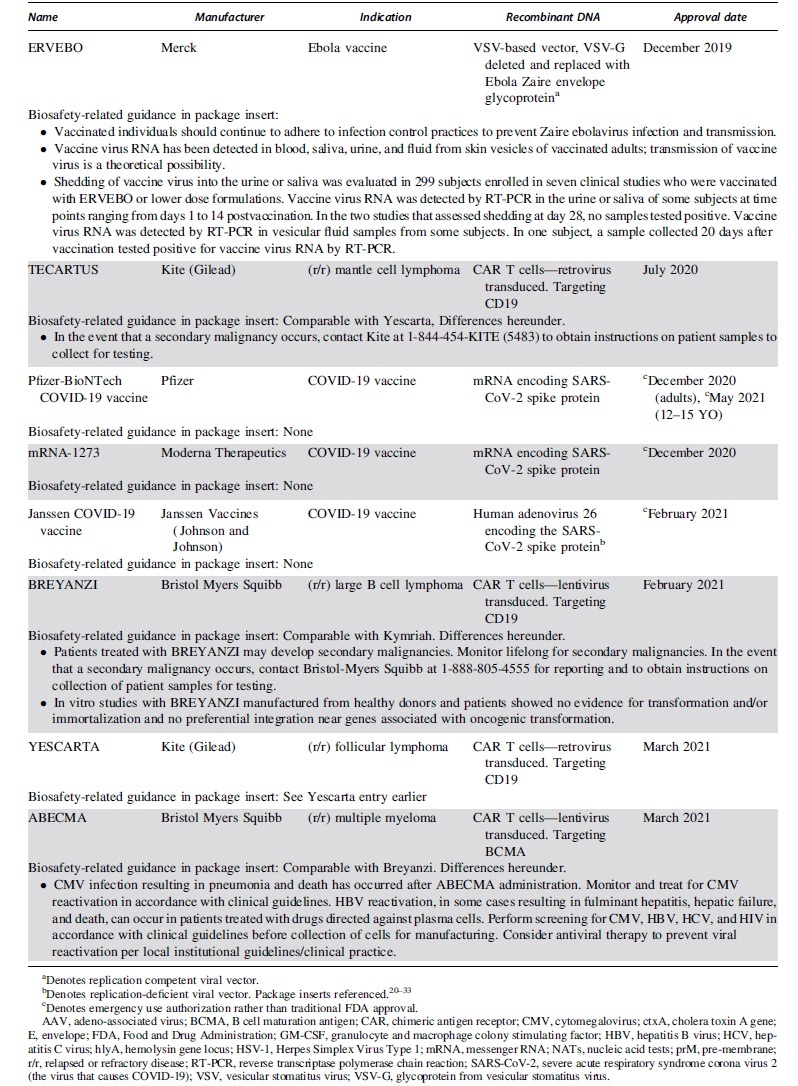

When the products are categorized by disease indication, we can gauge the level of progress gene therapy has made in each type of disease. Seven of the eight approved oncology products are chimeric antigen receptor (CAR) T cells against B cell malignancies. Six FDA approvals have been issued for infectious disease vaccines, although four of them are emergency use authorizations for vaccines against SARS-CoV-2 intended to quell the COVID-19 pandemic. Two adeno-associated virus (AAV)-based gene transfer vectors have been approved for use in diseases caused by rare inherited mutations. Luxturna was approved for subretinal injection to treat retinitis pigmentosa, a disease caused by mutations to the RPE65 gene that causes night blindness in children and progress to complete blindness in adolescence. Zolgensma was approved to treat spinal muscular atrophy, a neuromuscular disease caused by mutations to the SMN1 gene that typically results in children being unable to control the muscles required for breathing and dying by 2 years of age.

Six FDA-approved products comprise viral vectors. AAV-based vectors are currently the only FDA-approved gene transfer vectors intended to treat diseases caused by rare inherited mutations. Viral vectors based on replication-deficient human adenovirus 26 as well as replication competent yellow fever strain 17D204 and the Indiana strain of vesicular stomatitis virus have been approved for use as infectious diseases vaccines.

FDA-approved drugs contain a package insert describing the drug, associated risks, and handling instructions. Requirements for the format and content of this document are outlined in 21 CFR 201. Table 1 lists information pertinent to biosafety professionals from the package inserts for all FDA-approved products containing recombinant or synthetic nucleic acid molecules. The information provided is not comprehensive, so drug and site-specific risk assessments are still prudent practices. More detailed methodology for mitigating risks in the clinical setting are discussed elsewhere.^34–36^

## Multiple Revolutions Taking Place Simultaneously

Unprecedented levels of success are simultaneously taking place in different disease indications. The major developments are summarized as follows.

### Revolution 1: Clinical Applications of mRNA-Based Technology

mRNA-based technology came to the forefront in the Spring of 2020 as the Pfizer/BioNtech and Moderna COVID-19 vaccines were among the first to enter clinical trials. Moderna Therapeutics was the first to publicize the speed by which these vaccines could be produced. Table 2 shows the timeline of events for the research, development, testing, and approval of Moderna's mRNA-based vaccine, and compares it with the timeline for Janssen's (Johnson and Johnson) adenovirus-based vaccine.^37–45^ Whereas research and development of a vaccine can last years or decades, Moderna and the National Institutes of Allergy and Infectious Diseases collaboratively developed a vaccine in 2 days once the SARS-CoV-2 sequence was posted online. Within 6 weeks Moderna was able to produce and ship the first batch of vaccine for testing at the NIH.

**Table 2.** Comparison of timelines for the research, development, testing, and approval of Moderna's messenger RNA-based vaccine and Janssen's adenovirus-based vaccine against SARS-CoV-2

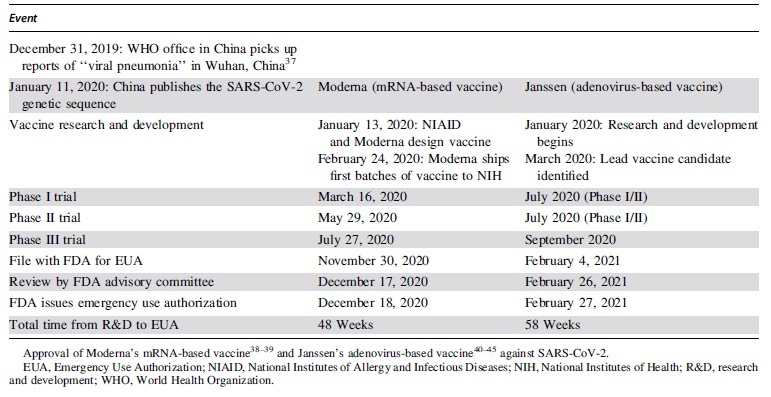

Whereas the speed with which mRNA vaccines can be developed and produced was remarkable, the efficacy was equally impressive as both Moderna and Pfizer's clinical trial data both showed efficacy at ∼95%.^46^ The first vaccines to receive FDA emergency use authorization were both mRNA based. As of this writing (July 5, 2021), 67.1% of adults in the United States have received at least one dose of a COVID-19 vaccine, 96% of which were mRNA-based vaccines.^5^

When interviewed on COVID-19 vaccines, Dr. Peter Marks said, “I think anyone involved in vaccinology, when we saw how effective these mRNA vaccines were against COVID-19, it was a surprise. We were expecting them to be ‘okay effective,’ but to see them so effective across the entire age spectrum, the different ethnic and racial backgrounds, was one of the most pleasant surprises in an otherwise bleak year.”^47^

The success of mRNA-based vaccines as well as the previously mentioned recombinant virus-based vaccines create hope for a new era of vaccinology.^48–50^ Rather than depending on traditional and time-consuming culture-based methods for developing and manufacturing vaccines, this new era utilizes recombinant DNA technology to create designer vaccines intended to utilize the bodies' cells to make the targeted antigen rather than manufacturing it in a factory. The speed with which vaccines have been developed, tested, mass produced, and distributed during the COVID-19 pandemic is unprecedented and bodes well for a new era on the war against infectious diseases.

Already as the COVID-19 pandemic begins to quell pharmaceutical companies are taking aim at their next ambitious targets. A recombinant vesicular stomatitis virus (VSV) - based vaccine against Ebola has already obtained FDA approval. Highly virulent agents such as Nipah virus are in the cross hairs for future vaccines. Vaccines against more common agents associated with high morbidity, including fetal pathogens such as Zika virus, herpes simplex viruses, and cytomegalovirus, have been developed and are approaching clinical trials.^51^ Newly developed vaccines against major causes of death in the developing world (respiratory syncytial virus, tuberculosis, HIV, and malaria) are also approaching clinical trials.^52–56^

With the success of mRNA-based vaccines, a number of large pharmaceutical companies are redirecting resources to focus on developing mRNA-based drugs for applications beyond infectious diseases.^50,57–61^ Oncology research in particular is shifting away from conventional treatment strategies that employ chemotherapy, radiation, or surgery and focusing on targeted immunotherapies.^62^ mRNA-based cancer vaccines are a promising alternative for a personalized treatment strategy designed to target tumor-associated antigens that are preferentially expressed in cancerous cells, such as growth factors or antigens that are unique to malignant cells arising from somatic mutation.^58–66^ The availability of high-throughput sequencing of tumor biopsies allows for the identification of neoantigens specific to the patient's tumor and the development of mRNA-based vaccines targeting them.^64,67,68^

### Revolution 2: CAR T Cells

CAR T cells have been successfully applied to treat resistant or refractory B cell leukemias and lymphomas. To date, the FDA has issued seven approvals for CAR T cell therapies and the number is likely to grow as hundreds of CAR T cell trials are currently taking place around the globe.^69–71^

**Figure 2.**
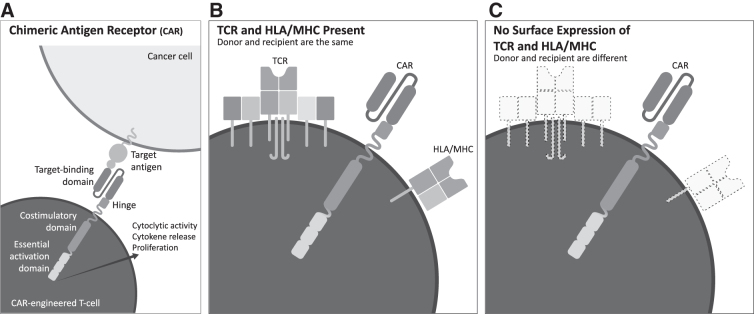
CAR T cells: **(A)** Common structure of a CAR. **(B)** Autologous CAR T cells express endogenous TCR and HLA/MHC. **(C)** “Off-the-shelf”/universal CAR T cells are modified to prevent surface expression of the endogenous TCR and HLA/MHC to avoid graft versus host disease and rejection of the CAR T cells, respectively. CAR, chimeric antigen receptor; HLA, human leukocyte antigen; MHC, major histo-compatibility complex; TCR, T cell receptor.

Gene transfer technology allows the modification of T cells to express alternatives to their endogenous T cell receptors. First-generation CARs comprised an extracellular antigen-binding moiety, a transmembrane domain and the intracellular CD3 zeta chain responsible for signal transduction from a T cell receptor (Figure 2A). The most common antigen-binding domain employed currently is a single chain variable fragment derived from the variable domain of antibodies. The CAR allows for binding of cell surface antigens without relying on antigen presentation within the context of the human leukocyte antigen (HLA). This approach is beneficial as downregulation of the HLA is a common means of immune evasion utilized by cancers. Once the CAR T cells bind their cognate antigen signaling through the CD3 zeta domain causes downstream T cell activation. Second- and third-generation CARs included costimulatory domains which enhanced T cell activation and had dramatic effects on efficacy as well as development of a memory phenotype that resulted in prolonged engraftment in vivo.

Autologous CAR T cells, where the donor and recipient are the same patient, typically require 2–4 weeks to progress from the initial leukaphereses product, through manufacturing and quality control testing before release for clinical use. As replication-deficient retroviral or lentiviral vectors are commonly used to transduce the T cells with the construct encoding the CAR, the final product must be tested for replication competent virus before release. Given the risk of insertional mutagenesis leading to oncogenic transformation, the FDA recommends a long-term follow-up period of up to 15 years for recipients of the cellular product.

Toxicities from CAR T cell therapies range from mild to severe and life-threatening, of note being cytokine release syndrome (CRS) and neurotoxicity. The severity of the CRS correlates with tumor burden. It is currently unknown if cerebral edema resulting from CAR T cell therapy is triggered by severe CRS or if a separate mechanism is at play. Study of the mechanisms involved is hampered by the lack of adequate animal models. However, both CRS and neurotoxicity are largely reversible with well-established treatment regimens. A description of the CAR T cell utilized as well as the associated risks and long-term follow-up period must be present in the IRB-approved informed consents utilized in clinical trials. CAR T cells have also been engineered with safety mechanisms to eliminate them in cases of severe toxicity such as drug-inducible apoptosis and constitutive expression of surface epitopes that can be targeted with FDA-approved monoclonal antibodies.

“Off-the-shelf” CAR T cell products derived from healthy donor T cells may mitigate several long-standing issues with autologous CAR T cell therapies.^72–75^ As the starting material does not originate from cancer patients prior conditioning from tumor-derived suppressor cells and T cell exhaustion are no longer a concern as well as insults from prior chemotherapy regimens. Off-the-shelf T cells are manufactured in advance and are intended to be readily available in a frozen state. Rather than requiring a patient to undergo leukapheresis and wait weeks for the manufactured product to be delivered for infusion, off-the-shelf CAR T cells only require thawing and transport to the infusion suite. Furthermore, the cells are manufactured from pools of healthy donors that allow for mass production and economies of scale to reduce costs rather than creating a single product for each patient with an autologous approach.

Gene editing technology makes off-the-shelf CAR T cell products possible (Figure 2B, 2C). Surface expression of the endogenous T cell receptor is prevented to avoid graft versus host diseases. Surface expression of the HLA can also be prevented to avoid host rejection of the engrafted cells and prolong their longevity in vivo. Prolonged engraftment of CAR T cells is associated with prolonged remission. Studies of autologous CAR T cells have seen engraftment lasting several years. More recent data with newly developed off-the-shelf CAR T cells have shown engraftment lasting 6 months. Further study is required to determine if off-the-shelf CAR T cells devoid of HLA expression can survive in recipients as long as autologous CAR T cells.

CAR T cells can be engineered to express multiple receptors that allow for combinatorial antigen-sensing circuits.^76,77^ The T cells can be engineered to utilize Boolean logic for greater precision and avoid on target, off tumor effects such as attacking nonmalignant bystander cells expressing one of the antigens targeted by the CAR T cells. This approach would require multiple different targeted antigens to be present on a cell before CAR T cell-mediated killing. Dominant negative or suppressive receptors can also be utilized to avoid attacking nonmalignant bystander cells.

Although CAR T cells have been greatly successful in treating B cell leukemias and lymphomas, they have had limited success in treating solid tumors. A likely cause for this phenomenon is the immune-suppressive tumor microenvironment. Designing CAR T cells for this purpose is an area of active study focusing T cell trafficking to the tumor, and abrogating the T cell's ability to accept suppressive signals whether they be contact dependent (PDL-1, CTLA-4, etc.) or independent (hypoxic environment, suppressive cytokine milieu, deprivation of T cell survival factors, etc.).^78^ Other approaches include employing other cell types such as CAR natural killer cells and CAR macrophages.

### Revolution 3: Multiple Options Abound for Developing Treatments for Rare Diseases

The 1983 Orphan Drug Act defines a rare disease as a disease or condition that affects <200,000 people in the United States.^79^ Over 7000 rare diseases affect >30 million people in the United States. Many rare conditions are life-threatening and most do not have treatments. The Orphan Drug Act creates incentives for development of orphan drugs to treat such diseases.^80^ Given the small number of individuals affected by any one rare disease or condition, a pharmaceutical company that develops an orphan drug may reasonably expect the drug to generate relatively small sales in comparison with the cost of developing the drug and consequently to incur a financial loss. Recent advancements in clinical use of recombinant DNA technology create opportunities for addressing great unmet medical need in the realm of rare diseases. “I am very excited for the field because I feel like we're beginning to get to a critical mass,” where a single method or product can be deemed safe and then adapted for many uses, said Dr. Peter Marks, head of the FDA CBER that regulates gene therapies.^81^

Two drugs based on AAV-based vectors have already received FDA approval for treatment of rare diseases, the aforementioned Luxturna and Zolgensma. AAV-based vectors are a popular tool for gene transfer to address single gene mutations.^82,83^ The basic biology of AAV vectors is summarized elsewhere.^84^ AAV is a small virus with a 4.7 kb single-stranded DNA genome giving it a limited payload capacity. Wild-type (WT) AAV is a Risk Group 1 organism that typically infects humans between 1 and 3 years of age. The resulting asymptomatic infection frequently results in both cellular and humeral immunity directed against capsid proteins.^85,86^ AAV vectors can infect both dividing and nondividing cells.

Whereas WT AAV may integrate into host genomes at specific AAV integration sites, AAV vectors devoid of the Rep gene tend to form episomes in the nucleus of transduced cells. Genomic integration is observed at 0.1–1% of transduction events, which has caused some concern about cancer risks. Whereas tumorigenesis was observed in a neonatal mouse model, it has not been observed in large animal studies or human trials.^87–89^ To date, 12 different AAV serotypes have been identified with capsids binding to different cellular receptors granting each serotype enhanced tropism for different cells and tissues.^84,90^ Whereas WT AAV is dependent on a helper virus for replication, commercially available packaging systems provide the necessary genetic material for viral replication in trans through multiple plasmids and enable creation of AAV vectors without utilizing a contaminating helper virus.^91^

Intravenous infusion of AAV results in trafficking to the liver, making AAV an attractive vector for hepatic gene therapy trials.^92^ Multiyear transgene expression after gene transfer to the liver has been observed in humans and large animals.^93,94^ Transgene expression in hepatocytes leads to antigen specific tolerance, which may be beneficial for diseases where intravenous infusion of therapeutic proteins leads to production of neutralizing antibodies that decrease efficacy.^95^

Although AAV is a popular gene transfer vector it has some drawbacks. Pre-existing neutralizing antibodies can interfere with the efficacy of the gene transfer vector when administered intravenously and may require administration of higher doses.^84,92,93^ Transgene expression takes place in a dose-dependent manner.^84,92,93^ Unfortunately, vector doses also correlate with anti-AAV capsid immune responses that can lead to elimination of transgene expressing cells. As AAV vectors are unable to replicate in host cells, transgene expression is diluted as transduced cells divide. Although AAV has traditionally exhibited an excellent safety profile, clinical trials utilizing high doses (e.g., 10^14^ viral genomes/kg), especially in subjects with pre-existing conditions, have caused some to express concern.^96–98^

Whereas AAV vectors provide long-term gene expression, mRNA-based technology is an attractive approach for developing therapies for rare monogenic diseases due to its transient expression profile.^99^ The transient nature of mRNA and high-safety profile allows for it to be utilized to restore or replace therapeutic proteins without relying on viral vectors. Delivery through lipid nanoparticle grants the mRNA access to the cellular cytosol leading to translation of the encoded protein without risks for insertional mutagenesis into the host cell genome. Reliance on cellular biosynthetic machinery allows the protein product to receive requisite post-translational modification and be trafficked to the appropriate subcellular compartment or be excreted based on the encoded leader sequence.

The mRNAs are not immunogenic eliminating concerns of neutralizing antibodies from repeated exposure to the gene transfer vehicle, which is a common concern with viral vectors. Unlike the permanent nature of viral vector-based gene transfer, mRNA is present transiently in cells limiting toxicity concerns. Owing to the nonpermanent nature of mRNA therapy, one has the ability to titrate the dose within an individual to identify an efficacious dose level and the ability to stop dosing in the event of an unexpected safety concern.

Packaging mRNA in lipid nanoparticles confers upon it sufficient environmental stability to be administered by injection into target tissues or nebulized for use in the respiratory tract for diseases such as cystic fibrosis. Nebulization creates the potential for droplets, aerosols, and contamination of the treatment area. A risk assessment should be performed to ensure adequate containment and decontamination strategies are in place.

## Considerations for IBC Review

The landscape for performing IBC review of clinical trials has changed considerably for the past few years.^1^ Gene therapy trials no longer require registration with the NIH OSP or consideration for review by the former NIH Recombinant DNA Advisory Committee. Appendix M of NIH Guidelines, which provided the bulk of guidance regarding IBC review of human gene transfer research, was removed from the 2019 version of NIH Guidelines. From that point forward, IBC review would be comparable with other types of nonexempt recombinant DNA and was covered under NIH Guidelines Section III-C-1. The documents previously required to register clinical trials with the NIH are now recommended for IBC review under the FAQ page titled Points to Consider for IBCs Reviewing Human Gene Transfer Protocols.^3^

IBCs sometimes face difficulties in obtaining the necessary information to perform a risk assessment and should take pre-emptive action to facilitate IBC review of clinical trials before their submission. IBCs overseeing research at academic medical centers should have the requisite expertise as well as codified policies and procedures for review of clinical trials. Guidance should be posted online detailing the documents required for IBC review. Clinical research personnel may have different backgrounds than laboratory research personnel and may be unfamiliar with the nature of IBC review. Unlike investigators in biomedical laboratories, clinical investigators typically do not create the investigational product containing the recombinant or synthetic nucleic acid molecules. As such, they are dependent on the study documentation sent to them from the pharmaceutical company serving as the research sponsor or the contract research organization (CRO) running the clinical trial. A detailed description of the design of the investigational product is typically found in the Investigator's Brochure (IB) in a section titled, Physical, Chemical, and Pharmaceutical Properties and Formulations. If the IB does not provide sufficiently detailed information for the IBC to perform a risk assessment, study personnel can request additional information from the sponsor or CRO. If study contacts are unable to answer the IBC's questions about the design of the investigational product, it may be beneficial to request the portion of the Chemistry Manufacturing and Controls (CMC) document detailing the design and manufacture of the investigational product.^100^ This document is required to obtain an IND from the FDA. The CMC document is routinely considered confidential and sponsors or CROs may be hesitant to divulge it without justification.

Clinical use of recombinant or synthetic nucleic acid molecules pose added challenges for IBCs and biosafety professionals. NIH Guidelines and the *Biosafety in Microbiological and Biomedical Laboratories* are written for research in the laboratory setting and more comprehensively address facilities for plant and animal research than the clinical setting. Once investigational products receive FDA approval confusion can arise regarding whether research oversight is required. On label use of approved drugs is not considered research and can be conducted without research oversight as standard of care. However, off label use of drugs containing recombinant or synthetic nucleic acid molecules brings those materials back under the IBC's purview for research purposes.

As the field of gene therapy matures and hundreds of studies progress toward late stage multisite trials, sponsors and CROs experience great efficiencies when utilizing independent, externally administered IBCs that are centrally administered. Centrally administered IBCs provide sponsors and CROs a single point of contact for submission, tracking and managing large multisite studies. Study-level documents are only submitted once and site-level personnel utilize the same forms, policies, and procedures across all sites to submit site-specific information (principal investigator, curriculum vitae, facility details, standard operating procedures, etc.). The efficiencies of centrally administered IBC review are comparable with those that drove the requirement for single IRB review of federally funded multisite clinical trials. A key benefit of single IRB review is the opportunity for faster testing of investigational products as well as increasing patient access to clinical trials that may result in life-altering or life-saving outcomes to individuals suffering from conditions with no approved treatment alternatives. Major academic medical centers are beginning to see centralized IBC review as necessary infrastructure for large-scale clinical trials.^101–103^ Yet, this important benefit of single IRB review can be lost because a similar mandate does not currently exist for biosafety reviews. Whereas approval from a central IRB may be issued in days, patient access can be delayed if IBC reviews are not completed just as quickly. This delay can be several weeks, when awaiting approval from an IBC that meets only once per month, or longer if the IBC requires multiple review cycles to issue approval.

## Conclusion

In this report, we highlight recent advancements in the field of gene therapy as well as some of the associated challenges that may be faced by biosafety professionals and IBCs with responsibilities over clinical research. We provide guidance in performing risk assessments for the currently approved gene therapy products as well as the most utilized types of investigational products in clinical trials. We also provide guidance for obtaining the necessary information to perform IBC reviews for these investigational products.

The COVID-19 pandemic has created several opportunities for continued growth of the gene therapy field. Efforts to vaccinate >70% of adults in the United States with vaccines containing recombinant or synthetic nucleic acid molecules will result in greater public acceptance of gene therapy research. Technological advancements that made the vaccine race possible will spur the next generation of gene therapy research. Advancements born in the developed world set the stage for the creation of therapeutics to treat greater numbers in the developing world and have the potential for massive benefits to global public health.

Biosafety professionals and IBCs have always played key roles in contributing to the safe evidence-based advancement of gene therapy research. Biosafety professionals responsible for clinical research oversight must be aware of the changing regulatory landscape as well as emerging technologies and their associated risks to support the safe and ethical conduct of research.
